# Novel Cell Therapy Using Mesenchymal Stromal Cell Sheets for Medication-Related Osteonecrosis of the Jaw

**DOI:** 10.3389/fbioe.2022.902349

**Published:** 2022-05-12

**Authors:** Nobuyuki Kaibuchi, Takanori Iwata, Yoko Kawase Koga, Toshihiro Okamoto

**Affiliations:** ^1^ Department of Oral and Maxillofacial Surgery, Tokyo Women’s Medical University School of Medicine, Shinjuku, Japan; ^2^ Institute of Advanced Biomedical Engineering and Science, Tokyo Women’s Medical University (TWIns), Tokyo, Japan; ^3^ Department of Periodontology, Graduate School of Medical and Dental Sciences, Tokyo Medical and Dental University (TMDU), Tokyo, Japan

**Keywords:** multipotent stromal cells, cell sheet, medication related osteonecrosis of the jaw, antiresorptive agent-related osteonecrosis of the jaw, periodontal ligament, cell therapy, anti cancer agents

## Abstract

Despite medication-related osteonecrosis of the jaw (MRONJ) being first reported in 2003, the optimal treatment and prevention modalities for MRONJ are not clear. As a result, dentistry, oral surgery, and departments involved in the treatment of cancer and bone diseases are struggling with the management of MRONJ. Several cases of MRONJ cannot be managed by conventional treatment strategies recommended in various position papers. Therefore, studies have been conducted to investigate the efficacy of novel therapies for MRONJ. However, the optimal treatment is unknown. Several cell therapies including autologous cell transplantation have been reported for MRONJ. Although the efficacy of cell therapy for MRONJ has been demonstrated, large, statistically accurate clinical trials are lacking. We have been investigating the efficacy of MRONJ treatment using mesenchymal stromal cell (MSC) sheets since 2013 and confirmed its efficacy through various experiments, wherein MSC sheets were transplanted in model rats and beagle dogs with MRONJ-like lesions. Based on these results, we are planning to conduct a clinical trial of MRONJ therapy using periodontal ligament-derived MSC sheets.

## Introduction

Bisphosphonates (BP) and anti-receptor activator of nuclear factor kappa-Β ligand (RANKL) antibodies (denosumab) are drugs that inhibit bone resorption by suppressing osteoclasts and are used in many patients with osteoporosis, bone metastases of cancer, and multiple myeloma. However, after the first report of BP-related osteonecrosis of the jaw in 2003 (Marx, 2003), reports of antiresorptive agent-related osteonecrosis of the jaw (ARONJ) have emerged worldwide. It has become a serious complication not only in dentistry and oral surgery but also in medical fields related to diseases of bone and cancer. In addition, osteonecrosis of the jaw has been reported with the sole use of some molecularly targeted drugs such as angiogenesis inhibitors, tyrosine kinase inhibitors, and tumor necrosis factor α (TNFα) inhibitors (Fleissig et al., 2012; Maluf et al., 2019). The American Association of Oral and Maxillofacial Surgeons has defined this disease as medication-related osteonecrosis of the jaw (MRONJ) (Ruggiero et al., 2014). The American Association of Oral and Maxillofacial Surgeons position paper has defined the following diagnostic criteria for MRONJ: 1) previous treatment with antiresorptive or antiangiogenic agents, 2) no history of radiation or bone lesions that are not cancer metastases to the jawbone, and 3) bone exposure in the oral, maxillary, or facial regions or presence of palpable bone through fistulas in or outside the oral cavity for more than 8 weeks. The disease has been staged from stages 1–3 according to symptoms. The pathogenesis, prophylaxis, and treatment are unclear, though many clinical and basic studies have been conducted. Many basic studies have suggested that the pathogenesis of MRONJ is suppression of bone metabolism, inhibition of angiogenesis, immune dysfunction, mucosal damage, infection by oral bacteria, and surgical invasion of the jawbone (Ruggiero et al., 2014; Yoneda et al., 2017). Since the effects of BP on the jawbone last for about 10 years (Russell et al., 2008) and MRONJ is associated with periodontal disease and dental implants, patients with osteoporosis and bone metastases of cancer are at risk of developing MRONJ for about 10 years after BP administration. Therefore, patients receiving BP require regular dental checkups and thorough oral hygiene maintenance. In addition, dental treatment or extraction of teeth before the administration of drugs that induce MRONJ can prevent MRONJ, and therefore, medical and dental cooperation is important (Yarom et al., 2019).

## Treatment of MRONJ

**FIGURE 1:**
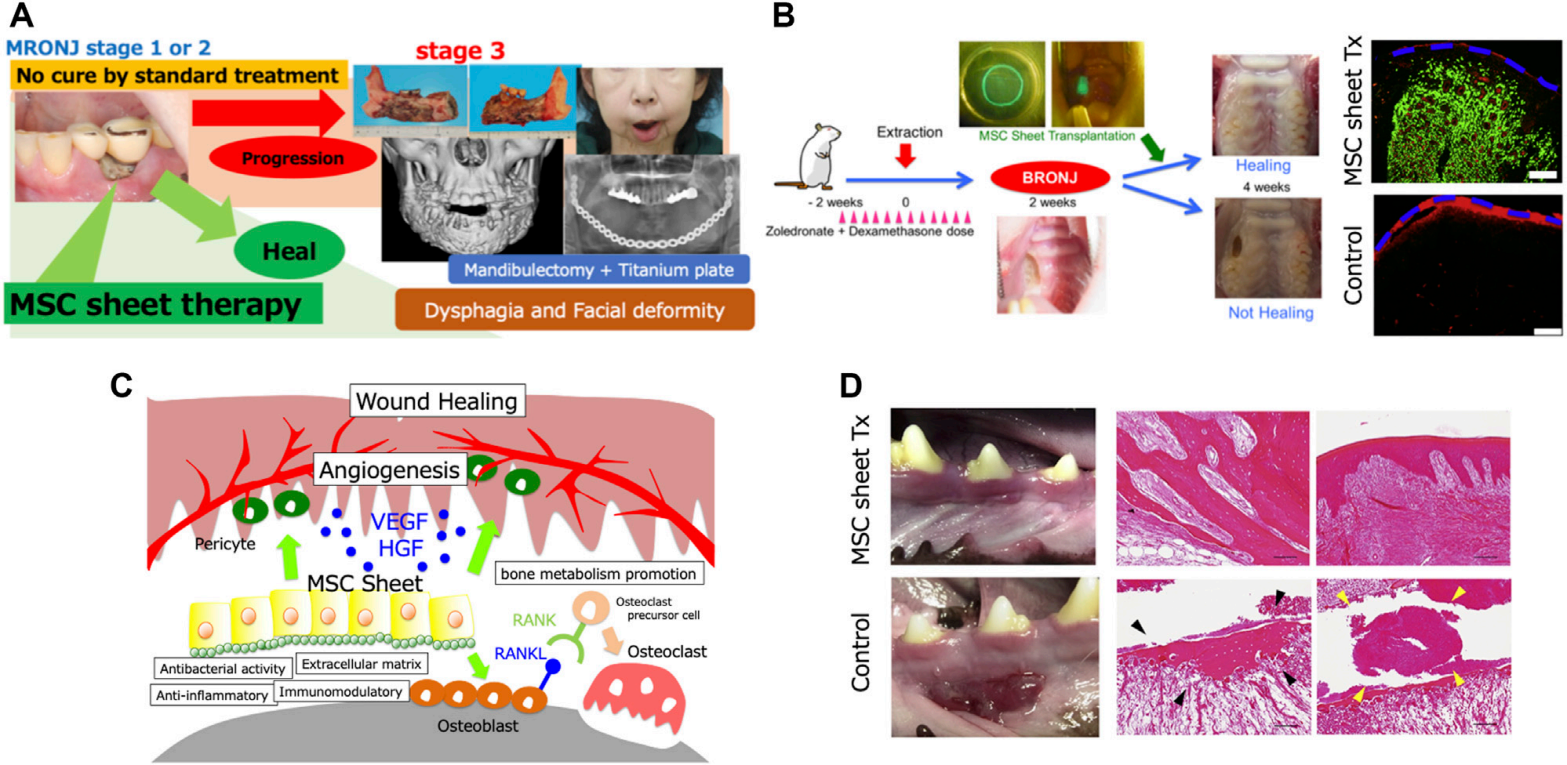
**(A)** The patient is a 64-year-old woman. She was administered with zoledronic acid and denosumab for breast cancer. Medication-related osteonecrosis of the jaw (MRONJ) developed around the mandibular dental implants and worsened to Stage 3, and underwent subtotal mandibulectomy. Due to the difficulty of reconstructive surgery, a titanium plate reconstruction of the mandible was performed. However, the titanium plate became infected and had to be removed. In such cases, a permanent tracheostomy may be necessary. **(B)** Transplantation experiments in a rat model. Healing of bone exposure was observed in the MSC sheet transplantation group, and significantly more submucosal neovascularization was observed. EGFP-positive cells (green) were observed in the transplanted sub-mucosa of the MSC sheet group at 2 weeks after transplantation. Furthermore, immunohistochemical analysis showed that these cells were localized around rat endothelial cell antigen-1-positive blood vessels (red). The blue dotted line indicates the mucosal surface. Scale bar: 200 μm. **(C)** Therapeutic effect of MSC sheets on MRONJ could be attributed to the combined effects of angiogenesis promotion by paracrine effects such as vascular endothelial growth factor and hepatocyte growth factor secretion and differentiation of MSCs into pericytes, bone metabolism promotion by osteoblast differentiation and RANKL expression, and the antibacterial, anti-inflammatory, and extracellular matrix effects of MSCs. **(D)** Transplantation experiment on a beagle dog model. Normal healing was observed in the MSC sheet transplantation group, while MRONJ-like findings such as mucosal inflammation, free sequestrum (black arrow), and bacterial aggregates (yellow arrow) were observed in the non-transplantation group. Scale bar: 100 μm.

The American Association of Oral and Maxillofacial Surgeons position paper advocates treatment according to the stage of the disease. For cases with mild symptoms (stages 1 and 2), conservative treatment such as administration of antibiotics, debridement, and mouthwash use is recommended. In cases progressing to stage 3, surgery to remove the jawbone is recommended. However, the cure rate after this treatment protocol is low. Rupel et al. reported a cure rate of 33% for stage 1 and 24% for stage 2 (Rupel et al., 2014) MRONJ. In recent years, early surgery with extensive resection of the jawbone even in cases with stages 1 or 2 disease has been advocated, and several studies have reported favorable results compared with conventional conservative treatment (Fliefel et al., 2015; Khan et al., 2015; Hayashida et al., 2017). When the disease progresses to stage 3, the only fundamental treatment option is the removal of the jawbone. However, it will inevitably result in a significant loss of the patient’s quality of life because of feeding, swallowing, and speech disorders and facial deformities (Figure 1A). In addition, the invasiveness of the surgery itself may lead to a recurrence of MRONJ. Therefore, the most appropriate therapeutic strategy is to cure the disease when it is in stages 1 and 2. Since there are many cases in which treatment provision according to the guidelines in the position paper has not resulted in cure, new treatment methods are being investigated. Hyperbaric oxygen therapy, which was used for the treatment of osteomyelitis and osteoradionecrosis of the jaw, was considered for MRONJ. However, a randomized controlled trial was conducted to investigate the efficacy of hyperbaric oxygen therapy for MRONJ, but it failed to show effective results (Freiberger, 2009; Freiberger et al., 2012). In contrast, a parathyroid hormone preparation, teriparatide, which is used for the treatment of osteoporosis and has a mechanism of action different from that of BP and denosumab, has been reported to be effective in randomized controlled trials. However, its use is limited because it is contraindicated in cancer patients and the duration of administration is limited to 2 years. In addition, the efficacies of various drugs and therapies such as platelet plasma, low-power laser irradiation, bone morphogenetic protein, pentoxifylline, and tocopherol have been investigated, but none of these have been clearly proven effective or widely implemented (Kaibuchi et al., 2021).

## Cell Therapy for MRONJ

Cell therapy has been investigated as a new treatment modality for MRONJ, and eight studies have been reported on the efficacy of cell transplantation in animal models of MRONJ (Table 1) (Barba-Recreo et al., 2015; Ogata et al., 2015; Kaibuchi et al., 2016; Kuroshima et al., 2018; Kaibuchi et al., 2019; Kuroshima et al., 2019; Zang et al., 2019; Rodríguez-Lozano et al., 2020). Most of these studies involved the allogeneic transplantation of bone marrow- or adipose-derived mesenchymal stromal cells (MSCs). In addition, cell supernatants of MSCs have been administered in animal models (Ogata et al., 2015). All studies showed that cell therapy was effective for MRONJ. In addition, 12 case reports of cell transplantation in patients with MRONJ in clinical practice have been documented (Table 1) (Bouland Cet al., 2020; Cella et al., 2011; Gonzálvez-García et al., 2013; Voss et al., 2017; De Santis et al., 2020). The transplanted cells were either autologous bone marrow cells or adipocytes, and cells are not used alone, but in combination with some scaffolds or artificial materials. In all cases, MRONJ was cured or improved. Therefore, cell therapy has been reported to be effective for MRONJ in clinical practice.

**TABLE 1:** Cell therapy for medication-related osteonecrosis of the jaw Animal studies.

Reference	Country	Source of Cells	Graft Type	Culture	Induction	Route	Other Materials	Animals
Animal studies								
Rodrí guez-Lozano et al. (2020), FJ et al. (2020)	Spain	Bone marrow-derived MSC	Allograft	Yes	No	Local	β-TCP	Rat
Kuroshima et al. (2019)	Japan	Peripheral blood mononuclear cells	Autograft	Yes	No	Intravenous	None	Mouse
Zang et al. (2019)	China	Human ASC	Xenograft	Yes	No	Local	Coral HA	Rabbit
Kaibuchi et al. (2019)	Japan	ASC	Allograft	Yes	No	Local	PGA sheet	Beagle
Kuroshima et al. (2018)	Japan	SVF cells	Allograft	No	No	Intravenous	None	Mouse
Kaibuchi et al. (2016)	Japan	Bone marrow-derived MSC	Allograft	Yes	No	Local	None	Rat
Ogata et al. (2015)	Japan	Media from human MSC	—	—	—	Intravenous	None	Rat
Barba-Recreo et al. (2015)	Spain	ASC	Allograft	Yes	No	Local	PRP	Rat
Li Y et al. (2013)	China	Bone marrow-derived MSC	Allograft	Yes	No	Intravenous	None	Mini pig
Kikuiri T et al. (2010)	United States	Bone marrow-derived MSC	Allograft	Yes	No	Intravenous	None	Mouse

Reference	Country	Source of Cells	Graft Type	Culture	Induction	Route	Other Materials	Study Design	Patient #
Clinical trials									
Bouland C et al. (2020)	Belgium	SVF	Autograft	No	No	Local	L-PRF	Case report	2
De Santis et al. (2020), GC et al. (2020)	Brazil	Bone marrow derived MSC	Autograft	Yes	Yes	Local	DBBM	Case report	2
Voss et al. (2017), PJ et al. (2017)	Germany	Bone marrow stem cells	Autograft	No	No	Local	Collagen membrane	Case report	6
Gonzálvez-García et al. (2013)	Spain	Bone marrow stem cells	Autograft	No	No	Local	PRP, β-TCP and DBM	Case report	1
Cella et al. (2011)	Italy	Bone marrow stem cell	Autograft	No	No	Local	Gelatin Spong	Case report	1

MSC, mesenchymal stem (stromal) cells; ASC, adipose-derived stem (stromal) cells; SVF, stromal vascular fraction; β-TCP, beta tricalcium phosphate; HA, hydroxyapatite; PGA, polyglycolic acid; PRP, platelet-rich plasma; PRF, plasma rich in growth factors; DBBM, deproteinized bovine bone mineral; DBM, demineralized bone matrix.

## MSC Sheet Therapy for MRONJ

Since 2013, we have been focusing on the effects of MSCs on wound healing, angiogenesis, bone metabolism, apoptosis, and immunomodulation to develop a new treatment modality for MRONJ using MSCs. Although intravenous administration is widely used as a method of transplantation of MSCs, most of the transplanted MSCs fail to reach the affected area, pulmonary embolization (Cyranoski, 2010) can occur, and cancer cell growth and metastasis can be promoted in cancer patients (Karnoub et al., 2007). In our experiments, we confirmed that MSCs did not attach to the affected area when administered intravenously (Kaibuchi et al., 2016). We considered the application of “cell sheet engineering” as a cell transplantation method that can overcome these problems. Cell sheet engineering allows cells to be collected in a sheet form, while retaining their extracellular matrix, by culturing them on a temperature-responsive culture dish (Yamato and Okano, 2004). The technique is being clinically used in various diseases, and its safety and efficacy have been confirmed (Nishida et al., 2004; Jonas et al., 2016; Miyagawa et al., 2017; Iwata et al., 2018). In addition, since the oral cavity can be easily approached, cell sheet transplantation can be performed in a minimally invasive manner.

We have investigated the efficacy of MSC sheet transplantation for the treatment of MRONJ in rats and beagle dogs (Kaibuchi et al., 2016; Kaibuchi et al., 2019). Before performing the transplantation experiments in animals, we examined the effects of BP on the transplanted cells. The MSCs collected from BP-treated rats showed a significant decrease in the secretion of vascular endothelial growth factor and gene expression of RANKL. Based on the results, considering the effects of BP on donor cells, the transplanted cells were obtained from animals that had not been administered BP. Thus, the experiment was performed as allogeneic transplantation. MSCs are known to be immune tolerant and can be transplanted without issues even in allogeneic cell transplantation (Aggarwal and Pittenger, 2005). By extracting the maxillary molars of rats treated with BP and dexamethasone, the rat MRONJ model was developed with a high probability. The transplantation of allogeneic bone marrow-derived MSC sheets in the rat MRONJ model by mucosal coating in the transplantation group after 2 weeks of transplantation resulted in significant healing of the MRONJ lesions (Figure 1B). Moreover, significantly more neovascularization was observed in the transplantation group than in the non-transplantation group, and the number of osteoclasts, which was reduced by BP administration, increased. Furthermore, some transplanted MSCs were positive for CD146, a marker for pericytes. These results suggest that the therapeutic effect of MSC sheets on MRONJ could be attributed to the combined effects of angiogenesis promotion by paracrine effects such as vascular endothelial growth factor and hepatocyte growth factor secretion and differentiation of MSCs into pericytes, bone metabolism promotion by osteoblast differentiation and RANKL expression, and the antibacterial, anti-inflammatory, and extracellular matrix effects of MSCs (Figure 1C) (Kaibuchi et al., 2016). We performed a similar transplantation experiment in beagle dogs. After the removal of the jawbone of a BP-treated beagle dog and implantation of allogeneic adipose-derived MSC sheets, inflammation of the mucosa was observed on the non-implanted side, whereas normal healing was observed on the implanted side. Histological examination showed that the non-implanted side exhibited free sequestrum and bacterial aggregates, an MRONJ-like finding, whereas the implanted side exhibited normal healing (Figure 1D).

## Discussion

The usual cell harvesting method using proteolytic enzymes destroys the adhesion proteins between the cells, so the cells are scattered. Therefore, it is difficult to transplant cells efficiently without the use of scaffolds. In contrast, cell sheet engineering is a technology that allows cells to be collected in a sheet form while retaining their extracellular matrix by using temperature-responsive culture dishes (Yamato and Okano, 2004). Therefore, cell sheets can be transplanted efficiently to the target sites without scaffolds. Whereas scaffolds used for cell transplantation carry a risk of infection, not using them reduces that risk. On the other hand, a disadvantage of cell sheets is that they need to be confluent until they achieve sufficient physical properties. Based on these considerations, the cell sheet is the appropriate method of cell transplantation for MRONJ.

The selection of a cell source for MSCs and the establishment of a cell bank of allogeneic (human) MSCs are essential for the clinical application of this therapy. We have established a bank of human periodontal ligament-derived MSCs (PDLMSCs) and conducted a physician-led clinical trial to investigate the safety and efficacy of human PDLMSC sheet transplantation for periodontitis. PDLMSCs have angiogenic, osteogenic, and immunomodulatory abilities comparable to those of MSCs derived from other tissues (Seo et al., 2004; Iwata et al., 2010; Yeasmin et al., 2014; Behm et al., 2019). Therefore, we hypothesized that PDLMSC sheet transplantation would be useful for the treatment of MRONJ. In addition, rather than constructing a new cell bank of MSCs to develop a treatment for MRONJ, which occurs in a relatively small number of patients, using MSCs from the cell bank established for the periodontal disease trial has advantages in terms of reduced employment of research resources and medical economics. Therefore, we are planning to conduct a clinical trial of MSC sheet-based therapy for MRONJ using PDLMSCs from the cell bank established in the periodontal disease trial.

## Conclusion

Currently, there is no established treatment or prevention method for MRONJ. We have demonstrated the efficacy of MSC sheet therapy for MRONJ in animal experiments. Based on the results, we are planning to conduct a clinical trial of MSC sheet therapy for MRONJ using PDLMSCs.

## Abbreviations

ARONJ, Anti-resorptive agent-related osteonecrosis of the Jaw; BP, Bisphosphonates; MRONJ, Medication-related osteonecrosis of the jaw; MSC, Mesenchymal stromal cell; PDLMSCs, periodontal ligament-derived MSCs; RANKL, Receptor activator of nuclear factor kappa-Β ligand; TNFα, Tumor necrosis factor-α.
